# Psychological meanings reported on access to guidance on love life and sexuality in prenatal consultations at a public primary health care service in a Brazilian metropolitan city: a qualitative study with pregnant adolescents

**DOI:** 10.1192/j.eurpsy.2024.1418

**Published:** 2024-08-27

**Authors:** P. E. Ortolan, M. P. P. Lipi, M. Borges, L. S. Valladão, R. A. Bastos, L. M. Guerra, D. B. A. P. Vale, E. R. Turato

**Affiliations:** ^1^ Lab of Clinical-Qualitative Research; ^2^gynecology and obstetrics, State University of Campinas, Campinas, Brazil

## Abstract

**Introduction:**

What topics from their personal lives do patients bring to talk to the clinical team, in addition to reporting their health-illness complaints, being examined, and receiving medical and nursing guidance? Knowing the symbolic aspects of the professional-patient relationship allows for care with more empathy and greater adherence to outpatient service follow-ups. The sociocultural contexts of vulnerable adolescents amplify the importance of reproductive health care and understanding perceptions about romantic relationships and sexuality. During adolescence, risky behaviours can interfere with life opportunities and the future. The lack of care for adolescents’ reproductive health is associated with irreparable physical and psychosocial consequences. In Brazil, the Unified Health System functions as an important support for the community.

**Objectives:**

To interpret the symbolic meanings attributed by pregnant teenagers regarding the possible experience of talking and receiving guidance about romantic relations and sexuality from the clinical team of public primary attention (in the EPA-2023, we presented the work “on family relationships”, another branch belonging to the same PhD research).

**Methods:**

We used the Clinical-Qualitative Method (Turato. Portuguese Psychos. J, 2000 2(1): 93-108). For data collection, the main researcher used the Semi-Directed Interview with Open-ended Questions In-Depth and Field Notes, fully transcript. The employ of the Seven Steps of the Clinical-Qualitative Content Analysis (Faria-Schützer et al. Cien Saude Colet. 2021; 26(1): 265-274) permits the solid discussion categories. Sample closed by saturation information criterion (Fontanella et al. Cad Saude Publica. 2008; 24(1): 17-27).

**Results:**

Sample of 10 adolescents, from 15 to 19 years. Interviewed by the first author, a female psychologist, online from September 2020 to June 2022. Findings validated by peer reviewers from Lab of Clinical-Qualitative Research. Categories to this congress: 1) maternal figure referred to as the axis of orientations on sexuality. This seems to empty the opportunity or the need to discuss these aspects in clinical consultations; 2) interviewees do not cite teenage pregnancy in its new emotional status as present in the prenatal consultation protocol; 3) there is a discourse of the re-signification of relationship with a loving partner by assuming also symbolically the pregnancy by both.

**Conclusions:**

Professionals that the teenagers access in clinical consultations at primary attention are not perceived psychoanalytically as transferential figures for conversations about romantic relationships and/or sexuality. It is opportune to rediscuss the expected and/or desired roles of the clinical team in that studied context from the psychic and cultural symbolic universe.

**Disclosure of Interest:**

None Declared

